# Application of a Reactive Agility Training Program Using Light-Based Stimuli to Enhance the Physical and Cognitive Performance of Car Racing Drivers: A Randomized Controlled Trial

**DOI:** 10.1186/s40798-022-00509-9

**Published:** 2022-09-05

**Authors:** Dávid Horváth, János Négyesi, Tamás Győri, Botond Szűcs, Péter János Tóth, Zsolt Matics, Csaba Ökrös, Sándor Sáfár, Nikolett Szabó, Beáta Takács, Róbert Kathy, Klára Tóth, David P. Ferguson, Ryoichi Nagatomi, Levente Rácz

**Affiliations:** 1 Department of Kinesiology, University of Physical Education, Budapest, Hungary; 2 Fit4Race Kft., Budapest, Hungary; 3 Division of Biomedical Engineering for Health and Welfare, Tohoku University, Sendai, Japan; 4 Department of Psychology and Sport Psychology, University of Physical Education, Budapest, Hungary; 5 PharmaFlight Research and Training Center, Debrecen, Hungary; 6 Deparment of Sport Games, University of Physical Education, Budapest, Hungary; 7 Training Theory and Methodology Research Center, University of Physical Education, Budapest, Hungary; 8 National Academy of Handball, Balatonboglár, Hungary; 9 Department of Kinesiology, Michigan State University, East Lansing, MI USA; 10 Department of Medicine and Science in Sports and Exercise, Tohoku University Graduate School of Medicine, Sendai, Japan

**Keywords:** Automobile racing, Cognitive performance, Driver science, Heart rate recovery, Maximal oxygen consumption, Motorsport, Vienna test system

## Abstract

**Background:**

There is a need to develop strategies that could contribute to the physical and mental preparation of motorsport athletes. A common method used by experienced motorsport athlete physical trainers is flashing light devices to train or assess reactive agility, despite limited evidence. Therefore, in the present study, we determined the effects of a 6-week reactive agility training program using light-based stimuli on the physiological and cognitive abilities of car racing drivers.

**Materials and Methods:**

The CONSORT guidelines for randomized controlled trial were used. In a single-blinded randomized controlled trial, 24 car racing drivers (EXP, *n* = 12; CON, *n* = 12) performed a comprehensive battery of cognitive tests marketed specifically at motorsport athletes from Vienna test system (VTS) at rest or during moderate intensity exercise on a bicycle. Physiological abilities were determined via a maximal incremental cardio-respiratory treadmill test. Baseline and post-intervention tests were performed on three consecutive days. Participants in EXP underwent a 6-week intervention consisting of 60-min training sessions twice a week using the Witty SEM light stimulus.

**Results:**

Participants in EXP but not in CON performed some of the VTS cognitive tasks with higher accuracy and/or shorter reaction time after the intervention at rest and during exercise. Car racing drivers performed the STROOP word-reading condition more accurately when the task was performed during the exercise vs. rest, regardless of group. In addition, the intervention induced beneficial changes in peak heart rate (HR), HR at gas exchange threshold, ventilation, and relative maximal oxygen consumption (rVO_2_ max). In contrast, body mass and fat mass increased, while peak HR and rVO_2_ max decreased in CON. Finally, participants in EXP improved their reactive agility performance and reaction time throughout the training program.

**Conclusion:**

Overall, the reactive agility training program using light-based stimuli appeared to be efficient to induce beneficial effects on some physiological and cognitive performance measures; therefore, it may have the potential to contribute to car racing drivers’ physical and mental performance.

**Supplementary Information:**

The online version contains supplementary material available at 10.1186/s40798-022-00509-9.

## Key Points

- The present article is the first to provide scientific evidence for the beneficial effects of a 6-week reactive agility training program using light-based stimuli on the physiological and cognitive abilities of car racing drivers.
- Cognitive abilities improved when performed at rest or during moderate intensity exercise on a bicycle ergometer. In addition, the intervention induced beneficial changes in physiological measures so that peak heart rate, the heart rate at gas exchange threshold, ventilation, and relative maximal oxygen consumption increased during the maximal incremental cardio-respiratory test.
- Our results suggest that such a short training program that is easy to administer has the potential of contributing to car racing drivers’ physical and mental performance.

**Supplementary Information:**

The online version contains supplementary material available at 10.1186/s40798-022-00509-9.

## Introduction

Prior to the year 2000, driving a racecar was not considered an athletic pursuit by the general population, mainstream media [1], and some exercise science/kinesiology researchers [2]. However, after 20 years of scientific investigation, racing drivers are now considered athletes [3–14]. Despite an increase in peer-reviewed investigations into the athletic attributes of racing drivers which documented the cardiovascular, environmental, cognitive and physical stressors (for review, see [15]), there is limited evidence regarding effective training regimes to improve motorsport athletes’ performance.

A potential reason for the lack of evidence-based training practices in motorsports is due to the dynamic nature of racing that merges physical and cognitive abilities of the driver with aerodynamic and mechanical engineering of the vehicle [16, 17]. Thereby, success on the racetrack cannot be reduced to a simple testable mechanism, but a combination of physical and mental attributes of the driver and engineering capabilities of the race team [15, 18, 19]. The Vienna test system (VTS) is a computerized tool for the assessment of sport psychology-related constructs in athletes, including the analysis of sustained attention, reaction time (RT), peripheral perception, stress reactivity, and time movement anticipation [20]. VTS is widely used by coaches, trainers, and practitioners to conduct cognitive testing in different sports, including motorsport (for review see [21]). Therefore, VTS could potentially be used to diagnose and/or develop cognitive skills relevant for racing performance.

Agility is one of the most important determinants of performance in many sports [22, 23]. It is defined as a rapid whole-body movement with a change of direction and/or velocity in response to an external stimulus [24]. The scientific literature can use the term ‘reactive agility’ to clearly distinguish this skill from the pre-planned change of direction agility movements [25, 26]. In traditional sports, coaches and researchers have used flashing light devices to produce external stimuli to which the participants needed to react [27–29]. However, these tools can be used not only to assess, but also to improve the reactive agility of the athletes. In motorsports, car racing drivers continuously need to respond correctly to several external stimuli during a race to have optimal performance. These stimuli include correct throttle, brake, and steering inputs to pilot the vehicle but also several visual stimuli to ensure the car is operating correctly. Many professional race cars have complex steering wheels that provide visual information (flashing lights) to the driver requiring them to depress a button or turn a dial. For example, in the Porsche 919 24 h of Le Mans winner, there were over 100 flashing light combinations that could appear on the steering wheel that required specific inputs from the driver. Therefore, we hypothesize that using flashing light devices that produce external stimuli to which the participants need to react may improve the reactive ability, and also the cognitive and physiological performance of car racing drivers. This hypothesis is supported by anecdotal examples presented on social media of elite racing drivers (e.g., *F*1) using such light stimuli to train or assess reactive agility, despite limited evidence.

Therefore, the aim of the present study was to determine the effects of a 6-week reactive agility training program using light-based stimuli on car racing drivers’ physiological and cognitive abilities. The training program was designed to model the complex physical and cognitive demands of motorsport competition in a controlled laboratory setting. We hypothesized that the training program would improve car racing drivers’ physiological and cognitive performance as compared to an inactive control group. Considering that car racing drivers have to perform under physiological strain during races, we hypothesized no differences in the results of cognitive tests when done at rest vs. during moderate intensity exercise. Moreover, we expected that the training program may further develop both abilities. Finally, we expected our participants in the experimental group to improve their reactive agility performance throughout the training program. Our study fits under the current efforts about gathering scientific data that could inform the motorsport community on proper training techniques.

## Methods

### Participants

Sample size calculations (G*Power 3.1.7 [30]) revealed that a minimum sample size of 20 participants would be appropriate to detect significant differences between the experimental and control groups, assuming a moderate effect size, type I error of 0.05, and power of 0.80. This study was a single-blinded randomized controlled trial. Participants were recruited through Fit4Race which has been involved in the preparation of racing drivers since 2014. Some of the participants were existing clients; however, they have never performed such cognitive tasks or practiced such agility training applied in the present study. The opportunity for participation in the study was advertised on the website and car racing drivers who met the criteria of the research were invited to the research facility. Participation was free of charge, the participants did not receive honoraria for the participation. Twenty-four car racing drivers (Rally, *n* = 12; Rallycross, *n* = 4; Touring car, *n* = 4; Formula 4, *n* = 4) volunteered in the study and were randomly assigned to either the experimental (EXP, *n* = 12; mean ± SD age = 24.7 ± 4.4 years; height = 181.4 ± 7.4 cm; mass = 79.3 ± 10.9 kg) or control (CON, *n* = 12; age = 25.6 ± 3.3 years; height = 178.8 ± 9.2 cm; mass = 78.8 ± 9.4 kg) group. Inclusion criteria were: (1) a minimum of 5 years of experience in racing at competitions, (2) participation at international championships in the previous season (year 2020)/achieving one of the top 5 ranks of the 2020th standings in the Hungarian national championship, and (3) no reported neurological deficits or sensorimotor impairment. After giving both verbal and written explanations of the experimental protocol, participants signed the informed consent document in accordance with the declaration of Helsinki. The study was carried out in accordance with the recommendations of the University Ethical Committee (Approval No. TE-KEB/No11/2020). The study was performed in accordance with CONSORT guidelines (Additional file 1: Appendix).

### Experimental Design

**Fig. 1:**
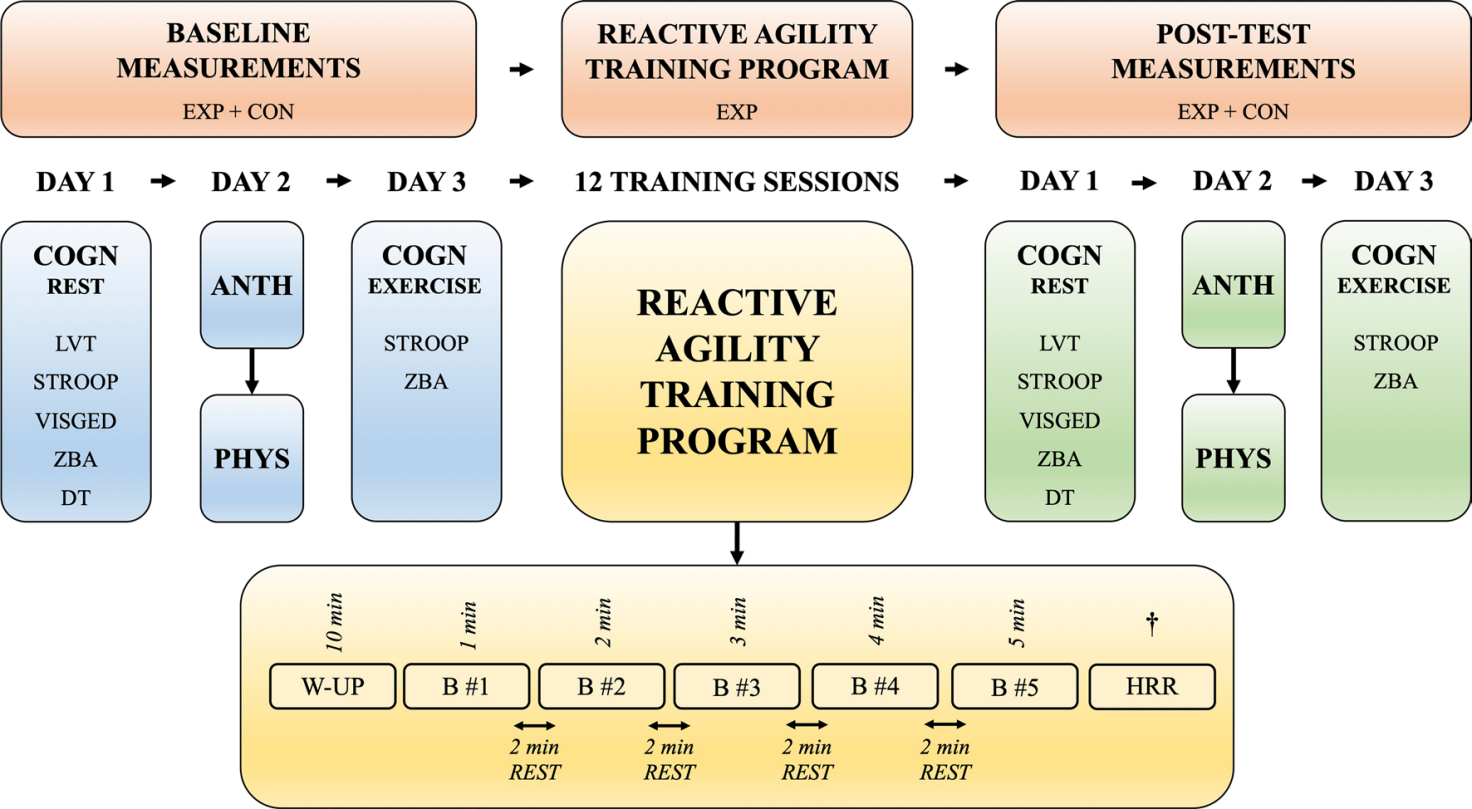
Schematic illustration of the experimental design. ANTH, anthropometric measurements; B, training block; COGN, cognitive measurements; CON, control group; DT, determination test; EXP, experimental group; HRR, heart rate recovery; LVT, visual pursuit test; PHYS, physiological measurements; REST, resting period between training blocks; STROOP, Stroop test; VISGED, visual memory test; W-UP, warm-up; ZBA, time/movement anticipation test. ^†^HRR was measured after the first, sixth, and 12th training bouts

**Fig. 2:**
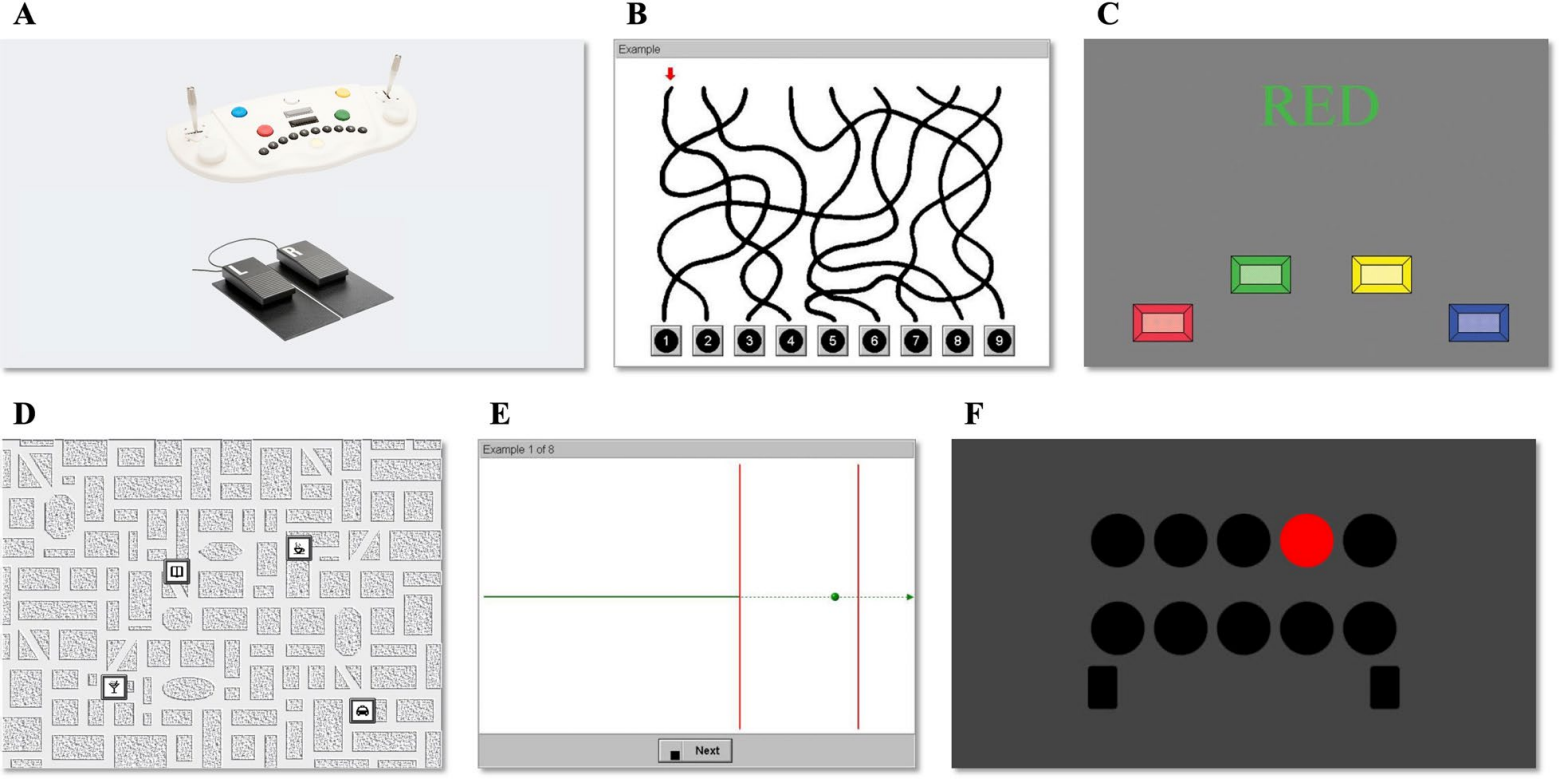
Vienna test system and representative examples of each cognitive task. **A** The Vienna test system (VTS). **B** Visual pursuit test (LVT): Participants followed the line marked with a red arrow out of nine intersecting arrows and found the right endpoint as quickly as possible. **C** Stroop test (STROOP): Participants were asked to press the appropriate button on the test panel as quickly as possible in two conditions (color naming, word reading). **D** Visual memory test (VISGED): Participants had to memorize and afterward recall the position of symbols on a city map. **E** Time/movement anticipation test (ZBA): A green ball appeared on the screen and moved at a certain trajectory with changing direction. At an unpredictable time, the ball disappeared and became invisible until two red lines appeared (**E**) from which the first line marked the place where the ball has disappeared. Participants were instructed to mark the anticipated position and time of the ball crossing the second red line using the buttons of the VTS panel. **F** Determination test (DT): The participants were presented with color stimuli and acoustic signals and were asked to react by pressing the appropriate buttons on the response panel

Figure 1 provides a schematic illustration of the experimental design. Participants had individual experimental procedures at the research facility (Fit4Race, Budapest, Hungary), which is specialized to test and train motorsport athletes. Both testing and training sessions were done in the morning and were controlled for Hawthorne effects [31, 32]. Specifically, D.H., R.S., or P.J.T. dressed in the official uniform of Fit4Race coaches and were always appropriately identified by their name written on their T-shirt, which also specified their status as ‘coach.’ Baseline and post-intervention tests were performed on three consecutive days. Participants were asked not to drink alcohol 24 h before and during the testing session and not to drink coffee in the mornings of the testing sessions. On the first day, participants performed cognitive tests on the computer. The cognitive tests consisted of a specific test package marketed for car racing drivers (SFMOTOR) from Vienna test system (VTS) including the visual pursuit test (LVT), Stroop test (STROOP), visual memory test (VISGED), time/movement anticipation test (ZBA), and determination test (DT) (Fig. 2). To eliminate auditory cues, a headset was provided during the experiment. The day after the cognitive tests, participants performed a maximal incremental cardio-respiratory test on a treadmill. The test was preceded by an anthropometry measurement in line with the international standards defined and approved by the International Society for the Advancement of Kinanthropometry (ISAK). Lastly, on the third day, participants performed the STROOP and ZBA tests during moderate intensity exercise on a bicycle according to the study from Torbeyns et al. [33].

The following week, participants in EXP began the 6-week training program twice a week with 48–72 h of rest in between each session. Each participant in both the EXP and CON groups was asked not to perform any cognitive- or motor skills development training during the research study. We also asked them not to change their daily routine or eating habits. Because the research was conducted during the offseason, EXP only performed the reactive agility training regime, while CON only performed their regular aerobic physical activity including walking and/or cycling. The post-tests were performed 2 days after the last training session and was identical to the baseline measurements.

### DAY 1: Cognitive Tests (Vienna Test System, VTS)

The Vienna test system (VTS) (Fig. 2A) is a widely used objective measure of various psychological constructs, which have the potential to provide information on the effects of certain factors on athlete cognitive performance (for review see [21]). In the present study, we selected and used five tests from a comprehensive cognitive test package of VTS marketed specifically at motorsport athletes called SFMOTOR (success factors in motorsport) to determine the effects of a 6-week reactive agility training program on car racing drivers’ cognitive performance. Each test was preceded by a familiarization (see the exact number of familiarization trials below for each test). The VTS system provided feedback to the participants in case of incorrect answers and did not allow them to proceed to the testing session until the correct answer was given.

#### Visual Pursuit Test (LVT)

The visual pursuit test was used to examine the visual orientation performance and selective visual attention of the car racing drivers by determining the perceptual quality of simple elements in a complex environment. After eight familiarization trials, participants started the LVT test. Specifically, an arrow appeared at the top of the display, pointing to the top of one of the nine curves, and the task was to indicate the number on the box to which that curve was connected as quickly as possible (Fig. 2B). The test took about 3–10 min. The median RT and the % of correct answers were evaluated.

#### Stroop Test (STROOP)

The Stroop test was used to examine the participants’ cognitive flexibility (or switching ability). The test is based on the assumption that the reading speed of a color word is slower if the word is written in a different colored font. Two conditions were used without interfering influences (congruent stimuli) to determine baseline performance and were related to the two interference conditions, i.e., (a) ‘color naming interference’ and (b) ‘word-reading interference’ (incongruent stimuli). Participants were asked to press the appropriate button on the test panel as quickly as possible (Fig. 2C). The test took about 10 min and was preceded by 10–10 familiarization trials for each condition. The reading and naming interference (the difference of the RT medians of the ‘interference conditions’ and the ‘baseline’) were evaluated and used for the statistical analysis. Besides the RT, the % of incorrect answers during the interference conditions was also analyzed to determine whether a faster RT was associated with more errors.

#### Visual Memory Test (VISGED)

The visual memory test was used to determine the visual short-term memory. Its value may provide information on how participants orientate themselves in a real-life environment. First, five familiarization trials were performed by the participants, and then they had to memorize and afterward recall the position of symbols on a city map (Fig. 2D). Specifically, the participant was initially presented with a city map on which four different symbols of locations (15 symbols in total, e.g., taxi station/hospital/school/church, etc.) are marked. Participants had to memorize the positions of the individual symbols and afterward recall them correctly. This was tested by presenting the city map without symbols and asking the participants to mark the location on the map where the symbol used to be by using a mouse attached to the VTS system. As soon as the respondent has marked a spot on the map, the actual position of the symbol in question is displayed, thus giving the respondent feedback on the correctness of his/her answer. The test took about 10–15 min. Total mean duration, total mean error duration, percent error duration (calculated as the ratio of total error duration to total duration), and coordination difficulty were used to create a score. A higher score indicates that the participant can remember a larger number of items of information.

#### Time/movement Anticipation Test (ZBA)

The time/movement anticipation test was used to determine participants’ skills in estimating the time-to-coincidence of an object to a designated point in space. The test was preceded by eight familiarization trials. A green ball appeared on the screen and moved at a certain trajectory with changing direction. At an unpredictable time, the ball disappeared and became invisible until two red lines appeared (Fig. 2E) from which the first line marked the place where the ball has disappeared. Participants were instructed to mark the anticipated position and time of the ball crossing the second red line. The position was determined by using the red and green buttons of the VTS panel that moved the cursor on the screen in both vertical (up and down, respectively) and horizontal (left and right, respectively) directions, while the timing of the crossing was determined by pressing the black rectangle button in the middle of the VTS panel (Fig. 2A). In the practice phase of the test, participants performed a practice consisting of 10 items, at which they received feedback. The test then contained 48 items, while no feedback was given. The test took about 5–25 min. The time and position error were evaluated as the time difference in seconds, and the deviation from the correct position, in pixels, respectively.

#### Determination Test (DT)

The determination test was used to evaluate participants’ reactive stress tolerance, attention, and reaction speed in situations requiring continuous, swift, and varying responses to rapidly changing visual, and acoustic stimuli. Before the start of the test, participants were given a practice session to familiarize themselves with the test. The participants were presented with color stimuli and acoustic signals and were asked to react by pressing the appropriate buttons on the response panel (Fig. 2F). Specifically, the color stimuli were presented via the monitor by (1) five different colors (white, yellow, red, blue, green) that could appear in one of the ten circles or (2) foot signals (left or right) to which the participants had to react as quickly as possible by pressing the corresponding colored button on the manual panel or one of the foot pedals of the VTS, respectively. The acoustic stimuli consisted of high and low tones that were presented via the Test System interface to which participants had to press the upper grey or the lower black rectangular button in the middle of the VTS panel, respectively. The stress element of the DT test comes from the adaptivity of the test, where the participants need to sustain continuous, rapid, and varying responses to rapidly changing stimuli. Adaptivity of the test means that the software continuously adapted the level of challenge based on the performance of each participant, i.e., the better the participant’s performance was, the faster the signals' presentation speed was. The test took about 6 min. The number of correct, incorrect, and omitted answers were evaluated and then were used in the statistical analysis.

### DAY 2: Anthropometric and Physiological Measurements

#### Anthropometric Measurements

Anthropometric parameters were measured according to the international standards defined and approved by the International Society for the Advancement of Kinanthropometry (ISAK). Specifically, height; body mass; width and depth of chest; shoulder, elbow, hip, and knee widths; body circumferences (chest, flexed upper arm, mid-upper arm, mid-lower arm, wrist, hand, thigh, calf, ankle), and skinfolds (biceps, triceps, subscapular, suprailiac, abdomen, mid-axilla, thigh, calf) were determined by a certified expert. Body mass (BM), body height (BH), and estimated muscle- (M%) and fat mass (F%) were measured and calculated, respectively.

#### Physiological Measurements

Participants performed a maximal incremental cardio-respiratory treadmill test until complete fatigue. The speed of the treadmill (Life Fitness model 90 T, Schiller Park, IL) was increased in the following manner: starting speed was 5 km/h with 0% inclination, which was increased linearly every second minute, to 10, 12, 14, and 16 km/h. Then inclination was increased by 3% every minute until exhaustion. A heart rate monitor (Polar H10, Polar Electro, Kempele, Finland) set at a sampling frequency of 1 Hz and a breath-by-breath metabolic cart (Jaeger Oxycon Pro, Viasys Healthcare GmbH, Höchberg, Germany) were used for measuring HR, VE, breathing frequency (BF), oxygen consumption (VO_2_), and carbon dioxide production (VCO_2_). V̇O_2_ max was defined as a lack of increase in oxygen consumption with increasing workload. The analysis of the straight-line relations of VCO_2_ vs VO_2_ (V-slope method) was used to detect the gas exchange threshold (GET) during the treadmill test [34]. The HR at GET (GETHR) was also recorded to detect the changes in HR corresponding to the lactate threshold.

### DAY 3: Cognitive Tests During Moderate Intensity Exercise

After a 5-min warm-up, participants performed the STROOP and ZBA tests (see details at ‘2.3 DAY 1: Cognitive tests (Vienna test system, VTS)’) during moderate intensity exercise (65–75% of peak HR) on a Precor upright bicycle (Precor, Woodinville, WA, USA). The response panel of the VTS was fixed on the ergometer aligned with the eyes of the participants. The experiment took ~ 25–30 min including the warm-up.

### Agility Training Program Using Light-Based Stimuli

The experimenters administering the training intervention were knowledgeable of the assigned group the participants were assigned. Participants in the EXP performed 60-min training sessions twice a week for 6 weeks. Participants performed a 10-min warm-up consisting of 2 min of walking (5 km/h) and 3 min of jogging (8 km/h) on a treadmill, which was followed by 5 min of dynamic stretching combined with core exercises.

The training bouts were performed using the Witty SEM^©^ visual reaction test device (Microgate, Bolzano, Italy). All testing and training sessions were performed in isolated rooms, in which the consistent temperature (20 °C) was regulated by a conventional air conditioner. The eight LED lamps were placed person-specifically in a 3 × 3 m dark room according to the results of the anthropometric measurements (Additional file 2: Fig. S1). Lamps ‘A’ and ‘B’ were placed in alignment with the tips of the shoulder, lamps ‘C’ and ‘D’ were placed on the height of the head with a wingspan between them, lamps ‘E’ and ‘F’ were placed on the mid-height of the shinbone with a wingspan between them, and lamps ‘G’ and ‘H’ were placed at the height of the waist with 85° from the starting position (+ 80 cm from arms reach). The starting position was determined as + 80 cm from the lamps and was marked on the floor with a tape (60 × 60 cm square). A green light was used as a stimulus, while the other photocells remained off. The test consisted of five series of 1 to 5 min of trials, respectively, with 2 min of rest in between.

Two sequences were pre-programmed in the following manner:

- Sequence #1: A,D,B,G,A,H,E,C,F,G,D,A,B,F,E,G,B,H,C,G,H,F,A,E,D,B,F,E,H,E
- Sequence #2: A,F,C,A,E,G,D,B,H,C,G,A,D,F,G,H,A,G,E,D,B,G,E,C,B,A,F,H,B,E

Sequence #1 was used for the blocks of 1, 3, and 5 min, while sequence #2 was used for the blocks of 2, and 4 min. The sequences were repeated until the end of the blocks. Participants were asked to wave only at the pre-specified photocell as soon as possible, i.e., block 1: green ‘B’; block 2: blue ‘6’; block 3: red ‘D’; block 4: green ‘9’; block 5: red ‘C.’ The generation time between the visual reactions was 1 s. The order of the lamps’ appearance and their position were randomly changed throughout the training program to prevent a potential learning effect of the sequences. HR data were measured using a Polar V800 heart rate monitor with a Polar H7 chest strap (Polar Electro, Kempele, Finland).

The reactive agility performance measured by the number of touches at each training and the RT were the primary performance outcomes. Average and peak HR were determined for each training. Moreover, parasympathetic reactivation was assessed after the first, sixth, and 12th training bouts from heart rate recovery (HRR) in a sitting position by taking the absolute difference between the final HR at exercise completion and the HR recorded following 60 s of recovery.

### Statistical Analyses

Statistical analyses were performed using SPSS Statistics Package (version 22.0, SPSS Inc., Chicago, IL). All data were checked for normal distribution using the Shapiro–Wilk’s test. When variables were not normally distributed, log-transformation was performed in each data within the corresponding analysis. These analyses were performed on the transformed data but the data are reported in the non-transformed form. To statistically investigate the effect of the 6-week training program on car racing drivers’ physical and cognitive performance and anthropometric measures, a series of a group (EXP, CON) × time (baseline, post) mixed analysis of variance (ANOVA) and post hoc tests were performed for each dependent measures. Separate group (EXP, CON) × time (baseline, post) × task complexity (at rest, during exercise) mixed ANOVAs were performed to determine if results of cognitive tests (STROOP, ZBA) are different when done at rest vs. during moderate intensity exercise. Additional repeated measures ANOVAs (rmANOVAs) and post hoc tests with Bonferroni correction for multiple comparisons were used to detect the changes in reactive agility performance, RT, and HR data (average HR, peak HR, HRR) of EXP throughout the training program. For each ANOVA, compound symmetry was evaluated with the Mauchly's test and the Greenhouse–Geisser correction was used when data violated the assumption of sphericity so that when the Epsilon was less than 0.75 for Mauchly’s test of sphericity, we used the Greenhouse–Geisser-corrected value and the Huynh–Feldt-corrected value for epsilon greater than 0.75. Effect sizes of repetition factors were expressed using partial eta squared (*η*_p_^2^) [35]. Complementary post hoc analyses (paired samples *t* tests) with additional Cohen’s effect size (*d*) calculation were used when indicated. Statistical significance was set at *p* < 0.05.

## Results

### Changes in Cognitive and Physiological Measures (Additional file 3: Data 1)

#### Cognitive Tests (Vienna Test System, VTS)

Table 1 summarizes the changes in each cognitive measure. Specifically, there was a main effect of time in LVT RT (*F*_1,22_ = 4.3, *p* = 0.05, *η*_p_^2^ = 0.16) and in the correct answers of LVT (*F*_1,22_ = 7.8, *p* = 0.011, *η*_p_^2^ = 0.26) with the post hoc analyses showing decreased RT and more correct answers post-intervention, irrespective of group. There was also a group × time interaction (*F*_1,22_ = 5.2, *p* = 0.033, *η*_p_^2^ = 0.19) in LVT RT. Post hoc analysis revealed that LVT RT decreased in EXP (*p* = 0.038, *d* = 0.61) but remained unchanged in CON (*p* = 0.800, *d* = 0.05) suggesting that the reactive agility training program enhanced car racing drivers’ visual orientation performance and selective visual attention.

**Table 1:** Reactive agility training program effects on psychological measures (Vienna test system)

	LVT		STROOP						VISGED	ZBA		DT		
			Naming			Reading								
	RT^†^ (sec)	Correct (%)	RT^†^ (sec)	Incorrect^†^ (%)	IF (sec)	RT^†^ (sec)	Incorrect^†^ (%)	IF (sec)	VMP (score)	TA (sec)	MDD (pixels)	Correct^†^ (score)	Incorrect (score)	Omitted^†^ (score)
*EXP*	*			*		*						*		*
BL	3.83 (1.31)	95.8 (5.8)	0.77 (0.17)	4.6 (2.6)	0.13 (0.09)	0.81 (0.16)	2.9 (1.9)	0.15 (0.09)	2.46 (1.05)	1.06 (0.49)	75.9 (35.0)	267.3 (32.9)	31.1 (15.3)	20.3 (6.1)
Post	3.19 (0.38)	100.0 (0.0)	0.68 (0.08)	2.2 (2.3)	0.10 (0.08)	0.72 (0.09)	1.5 (1.4)	0.13 (0.06)	3.04 (0.80)	1.17 (0.67)	72.2 (22.8)	288.8 (35.0)	23.5 (9.0)	15.5 (8.8)
*CON*			*				*					*		
BL	3.80 (0.58)	96.5 (4.2)	0.72 (0.10)	2.7 (1.8)	0.10 (0.06)	0.76 (0.08)	3.1 (2.4)	0.14 (0.05)	1.92 (1.90)	1.03 (0.48)	72.2 (19.8)	265.3 (48.1)	23.5 (12.8)	15.5 (7.9)
Post	3.80 (0.41)	97.3 (2.9)	0.76 (0.10)	3.3 (2.3)	0.13 (0.09)	0.83 (0.13)	3.3 (2.1)	0.21 (0.15)	1.84 (2.01)	1.11 (0.43)	72.4 (16.8)	259.7 (46.1)	26.3 (12.1)	17.2 (8.2)

Values are mean (SD) of each variables

BL, baseline; CON, control group; DT, determination test; EXP, experimental training group; IF, interference effect; LVT, visual pursuit test; MDD, median direction deviation; RT, median reaction time; STROOP, Stroop test; TA, time anticipation; VISGED, visual memory test; VMP, visual memory performance; ZBA, time/movement anticipation test

*p < 0.05 post hoc paired samples *t* test

^†^Significant group × time interaction

**Fig. 3:**
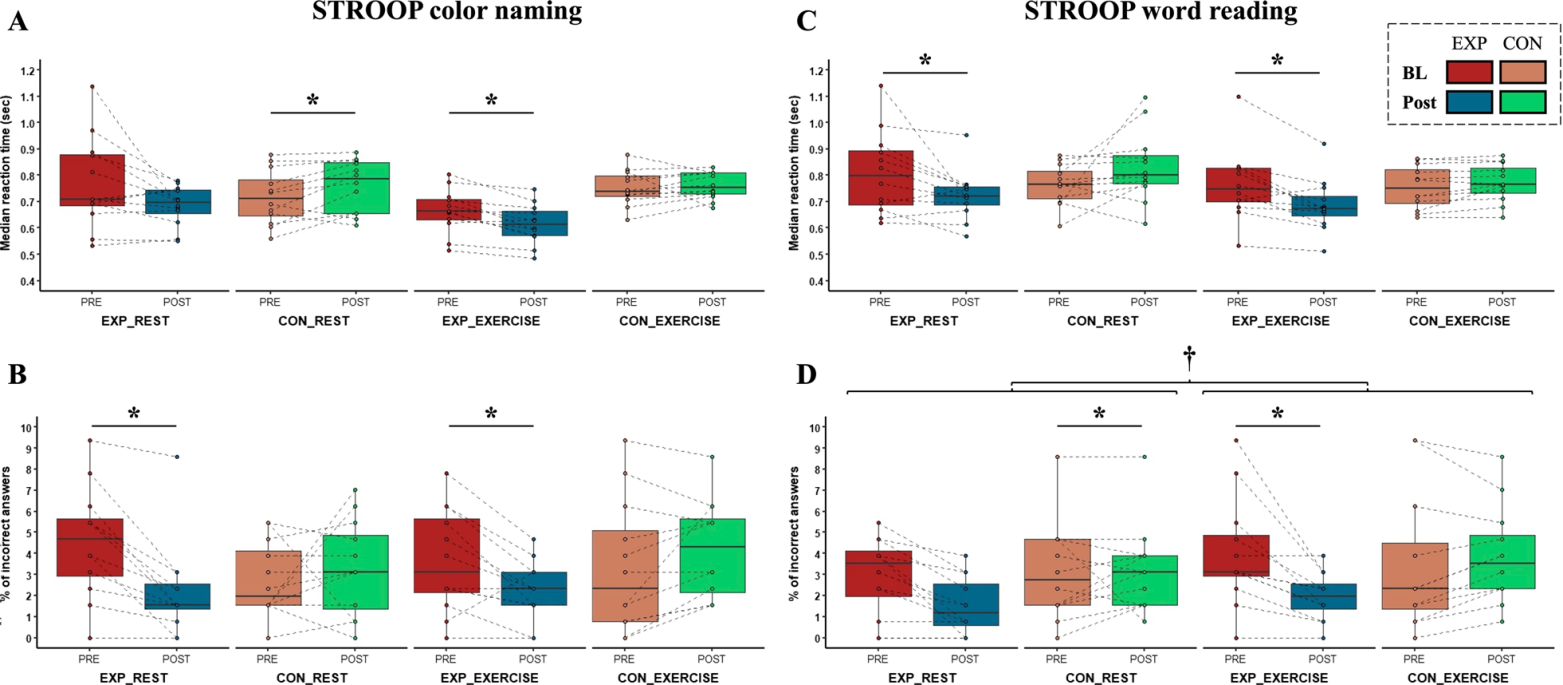
Changes in STROOP when performed at rest or during moderate intensity exercise. Baseline (BL) and post-intervention RT values in EXP (red and blue boxes, respectively) and CON (light brown and green boxes, respectively) in the color naming (**A**) and word-reading (**C**) conditions of STROOP. **B**, **D** represent the percentage of incorrect answers at baseline (BL) and post-intervention in EXP (red and blue boxes, respectively) and CON (light brown and green boxes, respectively) in the color naming and word-reading condition of STROOP, respectively. CON, control group; EXERCISE, STROOP during moderate intensity exercise; EXP, experimental group; REST, STROOP at rest; RT, reaction time; STROOP, Stroop test. The boxplots show the median, the upper, and lower quartiles, and the min and max value of the groups. ‘×’ within the boxplot represents the mean line. *p < 0.05 for post hoc paired samples *t* test based on a significant group × time interaction. †main effect of task complexity

A series of mixed ANOVA revealed a group × time interaction in both RT (*F*_1,22_ = 8.7, *p* = 0.007, *η*_p_^2^ = 0.28) and incorrect answers (*F*_1,22_ = 10.6, *p* = 0.004, *η*_p_^2^ = 0.32) of the color-naming condition of STROOP with the post hoc test showing a slightly increased RT in CON vs. EXP (*p* = 0.048, *d* = 0.05) (Fig. 3A) and decreased incorrect answers in EXP vs. CON (*p* < 0.001, *d* = 0.96) (Fig. 3B). Regarding the word-reading condition, a significant interaction in RT (*F*_1,22_ = 8.3, *p* = 0.009, *η*_p_^2^ = 0.27) was observed with a post hoc test showing that RT decreased in EXP (*p* = 0.028, *d* = 0.60) but remained unchanged in CON (*p* = 0.134, *d* = 0.56) (Fig. 3C). Moreover, a significant time main effect (*F*_1,22_ = 4.7, *p* = 0.041, *η*_p_^2^ = 0.18) and also a group × time interaction (*F*_1,22_ = 6.8, *p* = 0.016, *η*_p_^2^ = 0.24) was found in incorrect answers. Post hoc analysis revealed a decreased number of errors in CON (*p* = 0.021, *d* = 1), while EXP remained unchanged (*p* = 0.819, *d* = 0.09) most probably due to the large inter-subject variability (Fig. 3D) during the baseline measurements. STROOP interference effect was observed neither in color-naming nor in word-reading condition (all *p* > 0.05).

There were no time main effect or group × time interactions in VISGED and ZBA (all *p* > 0.05) indicating that the reactive agility training program did not induce any changes in participants’ visual memory performance and time/movement anticipation neither in EXP nor in CON. However, there was a time main effect (*F*_1,22_ = 6.4, *p* = 0.019, *η*_p_^2^ = 0.22) and also a group × time interaction (*F*_1,22_ = 18.5, *p* < 0.001, *η*_p_^2^ = 0.46) in DT correct answers with the post hoc test showing an increased score of correct answers in EXP (*p* = 0.05, *d* = 0.77) but a decreased score in CON (*p* = 0.04, *d* = 0.12) as compared to baseline values (267.3 ± 32.9, 265.3 ± 48.1, respectively) (Table 1). In addition, EXP but not CON also performed DT with a lower score of omitted answers indicated by a group × time interaction (*F*_1,22_ = 8.9, *p* = 0.007, *η*_p_^2^ = 0.29) and a post hoc paired samples *t* tests (EXP: *p* = 0.034, *d* = 0.78; CON: *p* = 0.087, *d* = 0.23).

#### Anthropometric Measures

Table 2 summarizes the changes in anthropometric measures in response to the reactive agility training program. Statistical analysis revealed that BM decreased in EXP (from 79.3 ± 10.9 to 77.2 ± 10.3 kg) but increased in CON (from 78.8 ± 9.4 to 79.7 ± 9.4 kg) indicated by a group × time interaction (*F*_1,22_ = 21.9, *p* < 0.001, *η*_p_^2^ = 0.50) with post hoc tests (*p* = 0.004, *d* = 0.20; *p* = 0.004, *d* = 0.10, respectively). Evidentially, BH remained unchanged in each group (all *p* > 0.05). Although no changes were found in M% (all *p* > 0.05), a time main effect (*F*_1,22_ = 47.3, *p* < 0.001, *η*_p_^2^ = 0.68) and also a group × time interaction (*F*_1,22_ = 70.4, *p* < 0.001, *η*_p_^2^ = 0.76) was found in F%. Post hoc analysis revealed lower post-intervention F% values in EXP (*p* < 0.001, *d* = 0.57) but higher F% in CON (*p* = 0.041, *d* = 0.05).

**Table 2:** Reactive agility training program effects on anthropometric and physiological measures

	Anthropometric measures				Physiological measures					
	BM^†^ (kg)	BH (cm)	M (%)	F^†^ (%)	HR^†^ (peak)	VE^†^ (l/min)	BF (BPM)	rVO_2_^†^ (max)	GETHR^†^ (bpm)	intensity (max)
*EXP*	*			*	*	*		*	*	
BL	79.3 (10.9)	181.4 (7.4)	43.9 (3.2)	18.6 (5.8)	194.3 (6.6)	129.3 (11.9)	55.8 (8.0)	49.9 (6.2)	170.9 (6.3)	15.5 (0.9)
Post	77.2 (10.3)	181.5 (7.4)	44.7 (2.3)	15.4 (5.5)	197.3 (5.9)	148.2 (11.4)	53.8 (7.6)	54.6 (4.7)	177.8 (5.7)	16.0 (0.0)
*CON*	*			*	*			*		
BL	78.8 (9.4)	178.8 (9.2)	46.0 (3.4)	15.8 (5.5)	195.0 (2.4)	126.6 (10.0)	51.8 (6.1)	50.3 (3.1)	172.9 (4.6)	15.3 (1.0)
Post	79.7 (9.4)	179 (9.2)	45.7 (2.8)	16.1 (5.6)	194.2 (1.5)	124.3 (8.1)	51.8 (6.1)	49.8 (2.8)	171.9 (3.7)	15.3 (1.0)

Values are mean (SD) of each variables

BF, breathing frequency; BH, body height; BL, baseline; BM, body mass; BPM, breaths per minute; bpm, beats per minute; CON, control group; EXP, experimental training group; F, estimated fat mass; GETHR, heart rate at gas exchange threshold; HR, heart rate; intensity, the peak treadmill speed reached (km/h); M, estimated muscle mass; rVO_2_, relative oxygen consumption (ml/min/kg); VE, ventilation

*p < 0.05 post hoc paired samples *t* test

^†^Significant group × time interaction

#### Physiological Measures

Table 2 summarizes the effects of the reactive agility training program on the physiological measures. Both peak HR and GETHR showed time main effects (*F*_1,22_ = 7.2, *p* = 0.014, *η*_p_^2^ = 0.25; *F*_1,22_ = 17.0, *p* < 0.001, *η*_p_^2^ = 0.44; respectively) and group × time interactions (*F*_1,22_ = 22.1, *p* < 0.001, *η*_p_^2^ = 0.50; *F*_1,22_ = 30.5, *p* < 0.001, *η*_p_^2^ = 0.58; respectively). Post hoc test revealed higher post-intervention peak HR values during the maximal incremental cardio-respiratory test for participants in EXP (*p* = 0.002, *d* = 0.45) and lower values in CON (*p* = 0.033, *d* = 0.40). In addition, GETHR was also higher in EXP as compared to baseline (*p* < 0.001, *d* = 1.2) but remained unchanged in CON (*p* = 0.086, *d* = 0.24). Mixed ANOVA revealed a time main effect (*F*_1,22_ = 25.4, *p* < 0.001, *η*_p_^2^ = 0.53) and also a group × time interaction (*F*_1,22_ = 43.1, *p* < 0.001, *η*_p_^2^ = 0.66) in VE with a post hoc analysis showing higher VE in EXP (*p* < 0.001, *d* = 1.62), which did not change in CON (*p* = 0.057, *d* = 0.28). Although BF remained unchanged (*p* > 0.05), there was a time main effect (*F*_1,22_ = 28.3, *p* < 0.001, *η*_p_^2^ = 0.56) and a group × time interaction (*F*_1,22_ = 41.5, *p* < 0.001, *η*_p_^2^ = 0.65) in rVO_2_ max. Post hoc analysis showed that rVO_2_ max increased in EXP (*p* < 0.001, *d* = 0.85) but decreased in CON (*p* = 0.029, *d* = 0.17).

#### Cognitive Measures During Moderate Intensity Exercise

The reactive agility training program induced changes in participants cognitive performance when performed during moderate intensity exercise on a bicycle, i.e., except for the interference effect, each STROOP variable showed improvement in EXP indicated by group × time interactions and post hoc analyses (Fig. 3). Specifically, there was a time main effect (*F*_1,22_ = 5.6, *p* = 0.027, *η*_p_^2^ = 0.20) and also a group × time interaction (*F*_1,22_ = 9.6, *p* = 0.005, *η*_p_^2^ = 0.30) in RT of the color-naming condition of STROOP. The post hoc test revealed shorter RT in EXP (*p* = 0.002, *d* = 0.75), which, on the other hand, remained unchanged in CON (*p* = 0.634, *d* = 0.01) (Fig. 3A). Moreover, the percent of incorrect answers also decreased in EXP indicated by a group × time interaction (*F*_1,22_ = 11.5, *p* = 0.003, *η*_p_^2^ = 0.34) and a post hoc paired samples *t* test (*p* = 0.015, *d* = 0.69), but did not change in CON (*p* = 0.084, *d* = 0.33) (Fig. 3B). Regarding the intervention-induced changes in word-reading condition, mixed ANOVA revealed a time main effect (*F*_1,22_ = 8.8, *p* = 0.007, *η*_p_^2^ = 0.29) and also a group × time interaction (*F*_1,22_ = 22.3, *p* < 0.001, *η*_p_^2^ = 0.50) in RT with a post hoc test showing shorter RT in EXP (*p* = 0.001, *d* = 0.66) but unchanged RT in CON (*p* = 0.063, *d* = 0.24) (Fig. 3C). In line with the results in the color-naming condition, car-racing drivers in EXP performed the word-reading task with higher accuracy (with less incorrect answers) indicated by both a time main effect and (*F*_1,22_ = 5.5, *p* = 0.028, *η*_p_^2^ = 0.20) also a group × time interaction (*F*_1,22_ = 13.8, *p* = 0.001, *η*_p_^2^ = 39) with post hoc test (*p* = 0.004, *d* = 1); however, the number of incorrect answers was not changed in CON (*p* = 0.224, *d* = 0.16) (Fig. 3D). STROOP interference interaction effect was not observed in any conditions (all *p* > 0.05), nevertheless, a time main effect was found in word-reading condition (*F*_1,22_ = 4.7, *p* = 0.041, *η*_p_^2^ = 0.18), irrespective of group. There were no time main effect or group × time interaction in ZBA variables (all *p* > 0.05) indicating that the reactive agility training program induced no changes in car racing drivers’ time/movement anticipation, regardless of group.

### Differences in STROOP and ZBA Performance when Performed at Rest or During Moderate Intensity Exercise

No significant group × time × task complexity or time × task complexity interaction effects were found in any of the cognitive task measures (all *p* > 0.05) indicating that the improvements in cognitive task performance did not differ when done at rest vs. during moderate intensity exercise in response to the training intervention, regardless of group. However, a significant main effect of task complexity (*F*_1,22_ = 8.0, *p* = 0.010, *η*_p_^2^ = 0.27) was found in the accuracy of the word-reading condition with the post hoc analysis showing more incorrect answers when the task was performed at rest vs. during moderate intensity exercise (*d* = 1.75) (Fig. 3D).

### Changes in Performance and Physiological Measures Throughout the Training Program

The light-stimuli-based reactive agility training program affected reactive agility performance markers but not physiological measures throughout the 6-week program (Additional file 4: Data 2). Specifically, statistical analyses revealed training main effects in both reactive agility performance (*F*_11,49_ = 15.4, *p* < 0.001, *η*_p_^2^ = 0.78) and RT (*F*_11,49_ = 38.2, *p* < 0.001, *η*_p_^2^ = 0.90) with post hoc analysis revealing that participants significantly improved from the first to the second, from the third to the fourth, and from the 11th to the last training (all *p* < 0.001) (Fig. 3A, B). Moreover, car racing drivers produced more touches with shorter RT in each training bout as compared to the first training (all *p* < 0.001), including the last 12th training, indicating an improved reactive agility performance in response to the training intervention. On the other hand, neither average- or peak HR nor HRR were affected throughout the training program (all *p* > 0.05).

## Discussion

This study is the first to examine the effects of a 6-week reactive agility training program using light-based stimuli on physiological and cognitive performances in car racing drivers. The main findings of the present study are that cognitive performance on some sub-tasks of the VTS improved in response to the intervention even when the tasks were performed during moderate intensity exercise on a bicycle. What is more, car racing drivers performed the STROOP word-reading condition more accurately when the task was performed during the exercise vs. rest. Furthermore, the training program-induced beneficial changes in physiological measures of car racing drivers in EXP, including higher peak- and gas exchange threshold-related heart rate, improved ventilation and relative maximal oxygen consumption during a maximal incremental cardio-respiratory test. Finally, car racing drivers performed the reactive agility task with improved performance and decreased reaction time throughout the 6-week training program. Overall, our results indicate that such a short training program that is easy to administer may have the potential to contribute to car racing drivers’ physical and mental performance.

### Changes in Cognitive Performance at Rest or During Moderate Intensity Exercise

The peer-reviewed literature indicates that car racing drivers have significantly faster RT as compared to controls [36, 37] that might be related to the more consistent recruitment of brain areas devoted to motor control and spatial navigation of car racing drivers [38, 39], which are linked to enhanced eye movement strategies acquired by task familiarity [40]. It is, therefore, not questionable that visual perception and visual attention play an important role in driving performance [41]. Notably, while the visual behavior of saccadic scanning is prevalent in everyday driving [42], car racing drivers must rely more on peripheral vision [43, 44]. Therefore, in the present study, it was fundamental to determine whether the reactive agility training program has the potential to enhance car racing drivers’ visual attention. Indeed, participants in EXP but not in CON had decreased reaction time of LVT task post-intervention (3.19 ± 0.38 ms) as compared to baseline (3.83 ± 1.31 ms), suggesting that the reactive agility training program enhanced car racing drivers’ visual orientation performance and selective visual attention (Table 1). Moreover, the 6-week intervention induced beneficial effects in the color-naming condition of STROOP indicated by a decrease in incorrect answers in EXP but not in CON (Fig. 3). Regarding the word-reading condition, a decreased RT in EXP was found. Against the expectations, a decreased number of errors was found not in EXP but in CON, however, this unexpected result could be related to the large inter-subject variability during the baseline measurements (Fig. 3). Although the reactive agility training program induced changes neither in car racing drivers’ time/movement anticipation measured by the ZBA test nor in their visual short-term memory measured by VISGED, car racing drivers in EXP had an increased score of correct answers and a lower score of omitted answers of DT as compared to baseline (Table 1). Car racing is a sport that requires minimal errors in physical or cognitive performance. However, unlike more traditional sports, the high rate of speeds drivers compete at can result in more substantial injuries when errors do occur [15, 17, 45]. The determination test (DT) is commonly used not only in healthy young [46] and older [47] adults, but also in brain-injured respondents [48] to measure complex choice reactions under stress arising from the need to react continuously and as quickly as possible to rapidly changing stimuli. In line with previous studies suggesting a beneficial role of aerobic endurance training to improve stress tolerance [45], findings of our study indicate that a reactive agility training program may also have the potential to enhance car racing drivers’ reactive stress tolerance, attention, and reaction speed.

In addition, because car racing drivers continuously need to respond to several external stimuli during a race, we aimed to evaluate the information-processing capacity of car racing drivers performing two tasks simultaneously. This idea is supported by many anecdotal examples presented on social media of elite racing drivers (e.g., *F*_1_) performing conditioning and cognitive tasks simultaneously. For this aim, participants had to perform the STROOP and ZBA cognitive tasks during moderate intensity exercise on a bicycle. In both STROOP conditions, a decreased RT and number of errors were found in EXP (Fig. 3). We hypothesized no differences in the results of cognitive tests when done at rest vs. during moderate intensity exercise. In line with our hypothesis, we found no group × time × task complexity or time × task complexity interaction effects indicating that the improvements in cognitive task performance did not differ when done at rest vs. during moderate intensity exercise in response to the training intervention. What is more, car racing drivers performed the word-reading condition more accurately when the task was performed during the exercise vs. rest. This result is in line with results of meta-analytical studies [49, 50] that suggested an inverted U-shaped relationship between exercise intensity and cognitive performance, i.e., moderate levels of exercise increased physiological arousal and facilitated cognition. Overall, our results indicate that the reactive agility training program may have the potential to improve car racing drivers’ selective attention and information-processing speed even during a concomitant task. Nevertheless, the STROOP interference effect was not observed in any conditions neither when performed at rest nor during moderate intensity exercise suggesting that response RT of car racing drivers improved not only during the incongruent but also during the congruent stimuli.

### The training program induced anthropometric and physiological changes

Body mass and F% of car racing drivers decreased in EXP but increased in CON suggesting a potential de-training effect in CON (Table 2). However, the low effect size might indicate that these changes have no practical significance to traditional sports, but in automobile racing where the car/driver combination is designed to have a minimal mass to improve performance any training regimen that reduces body mass could have a beneficial effect [10]. Since we did not control the nutritional intake of car racing drivers throughout the 6-weeks reactive agility training program, we cannot interpret whether the decreased values in body-fat-related anthropometric measures were the result of an inconsistent calorie intake or the training itself.

In the present study, we aimed to determine whether the reactive agility training program induces beneficial changes in physiological indices. Although statistical analysis revealed significantly higher peak HR values in EXP during the maximal incremental cardio-respiratory test as compared to the baseline, the average of 3 bpm change is most probably practically meaningless. In particular, because the literature suggests that peak HR is reduced following regular aerobic exercise by sedentary adults and endurance athletes and can increase only upon cessation of aerobic exercise (for reviews, see [51, 52]). Thus, the slight increase observed in peak HR can be explained by the possibility that participants might have failed to actually achieve their true ‘maximal’ heart rate prior to the agility training program; therefore, it is not inconceivable that an increase in peak HR could appear when the experiment consists of repeated measures.

The analysis of the straight-line relations of VCO_2_ vs VO_2_ (V-slope method) was used to detect the gas exchange threshold during the treadmill test [34]. Car racing drivers’ HR at gas exchange threshold (GETHR) increased from 170.9 ± 6.3 to 177.8 ± 5.7 bpm in response to the training intervention (Table 2). Considering that variations in HR during exercise correlate with changes in exercise intensity [53], our result indicates that GET occurred later, at a higher intensity during the maximal incremental cardio-respiratory test; therefore, the reactive agility training program induced improved lactate threshold. In line with this, this short training program that is easy to administer was sufficient to evoke beneficial changes in cardiorespiratory measures, i.e., ventilation and rVO_2_ max also increased after the 6-week reactive agility training program. To the best of our knowledge, previous studies have not investigated the effects of a reactive agility training program neither on maximal oxygen consumption nor on ventilation. Nevertheless, our results are in line with the findings of a previous study sponsored by the US Air Force Research Laboratory showing increased levels of rVO_2_ max in response to an agility training consisting of ladder drills, hurdle crossings, dot/footspeed drills, and directional change drills in subjects undergoing military technical training [54]. In summary, the reactive agility training program appeared to be sufficient to induce changes in cardiorespiratory variables.

### Changes in performance and physiological measures throughout the 6-weeks training program

**Fig. 4:**
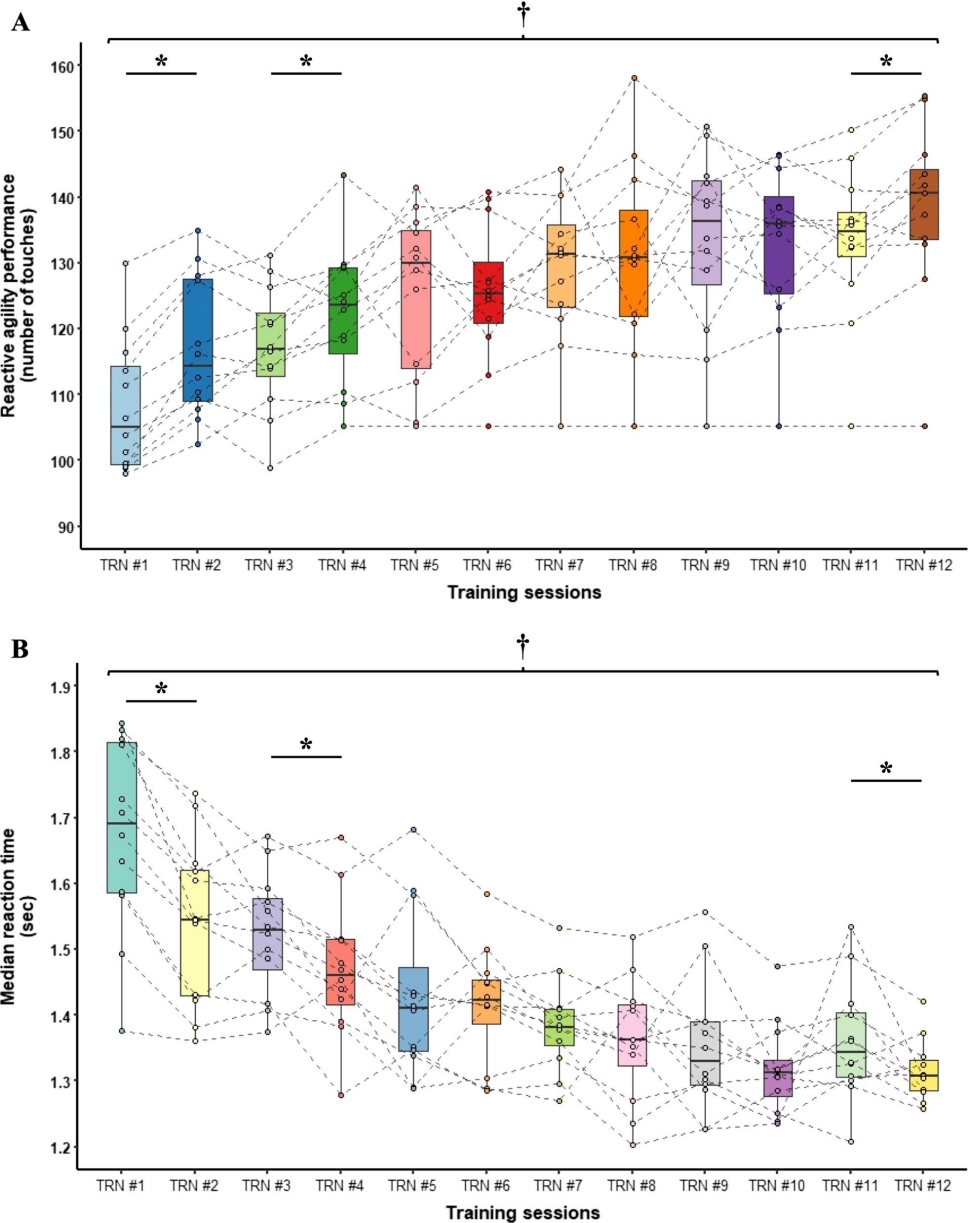
Changes in reactive agility performance and reaction time throughout the training program. **A** Participants significantly improved their reactive agility performance (**A**) and RT (**B**) from the first to the second, from the third to the fourth, and from the 11th to the last training. Moreover, car racing drivers produced more touches with shorter RT in each training bout as compared to the first training. *p < 0.05 for post hoc paired samples *t* test based on a significant time main effect. ^†^Significantly different to training session #1

The reactive agility training program using light-based stimuli was designed to model the complex physical and cognitive demands of motorsport competition in a controlled lab setting. Racing drivers respond to visual, physical (sliding of the car), and auditory (sound of the tires) signals in order to achieve optimal performance, thereby using reactive agility training may improve drivers’ abilities to prevent the incidence of catastrophic events. In the present study, the change in reactive agility performance was determined by measuring the number of touches on the flashing light devices to which the participants needed to react and also the time needed for the reaction. In line with the expectations, car racing drivers produced more touches with shorter RT in each training bout as compared to the first training (all *p* < 0.001), including the last 12th training, indicating an improved reactive agility performance in response to the training intervention (Fig. 4).

Exercise is known to induce acute and long-term responses in both heart rate (HR) and heart rate recovery (HRR) [55]; however, in the present study, neither average- or peak HR nor HRR was affected throughout the training program. The lack of HR adaptation might be simply explained by the lack of a sufficient training load. Although the reactive agility training program consisted of 60-min sessions twice a week for 6 weeks, the five blocks of reactive agility exercise lasted altogether only for 15-min (Fig. 1). It is, therefore, not surprising that the 15-min exercise together with the 10-min warm-up failed to induce changes in average and peak HR. Heart rate recovery may reflect the state of the autonomic nervous system, thus, may indicate the body’s capacity to respond to exercise [56]. Although endurance training is known to induce changes in cardio autonomic regulation [57], as mentioned before, the reactive agility training program applied in the present study cannot be considered as endurance training due to its insufficient training load. Nevertheless, considering that the number of touches and the speed of performing the reactive agility task increased throughout the training program, the unchanged HR may indicate a physiological improvement i.e., car racing drivers performed more work as the training program progressed with the same cardiovascular response. Overall, car racing drivers performed the reactive agility training with improved performance and decreased reaction time throughout the training program; however, the training load was insufficient to induce changes in heart-rate-related physiological measures.

### Limitations and future directions

In the present study, a comprehensive cognitive test package of VTS was performed. Although the package is marketed specifically for motorsport athletes, the manufacturer claims that 1) ‘The SFMOTOR test set emerged from a series of sport psychology studies conducted at the psychology faculty of the University of Vienna…’ and 2) ‘The findings of the studies led to the development of the SFMOTOR test set and definition of a target range for the ability profile of elite racing drivers,’ we found no peer-reviewed publication of these validation studies. However, since most of the tests are similar to standard cognitive psychology experimental tests that have been in use for decades to study both normal and clinical populations [58–62], the results provide useful information in regard to the cognitive performance of the participants. Nevertheless, some of the tests still have some limitations. First, the naming ‘visual pursuit test’ is misleading considering that ‘pursuit’ refers to tracking moving objects with the eyes; however, no eye movement behaviors are measured in this test. Second, the experimental setup of the VISGED test is not within the three general classes of tasks that have most often been used in humans to study visual short-term memory (Brook Matrix Task [63]; recall procedure of colored squares [58]; sequential comparison procedure of colored squares (e.g., change-detection task [64]); nevertheless, it can be considered as a modified version of the recall procedure of colored squares that was constructed primarily on the basis of Kosslyn’s theory of visual representation [59] and Hänggi’s integrative information processing model [60]. On the other hand, it is questionable how this test is ‘marketed for motorsport’ according to the manufacturer’s claim as most drivers do not need to look for a taxi or specific locations while racing. Moreover, researchers and practitioners using the ZBA test of the VTS should acknowledge that this test is commonly called a ‘prediction-motion task’ [61, 62] and not a time/movement anticipation test. Although the DT test is an adaptive test so that the software adjusts the stimulus presentation speed to the performance level of the participant, no detailed information is given regarding the exact algorithm of the level of the adaptation. Considering that these cognitive tests were designed for normal or clinical populations, it is still unknown whether elite athletes would also fit within the scope. Finally, while the number of familiarization trials was pre-programmed in the VTS software, we found no data or validation studies that would support whether the familiarization approach is appropriate and sufficient enough.

Another limitation of the present study is the lack of a double-blinded study design, i.e., the experimenters administering the training intervention were aware of which group the participants were assigned. Moreover, the 6-week training program in EXP was only controlled with an inactive CON group. Considering that our training program aimed to improve the reactive agility of car racing drivers, we should have had another control group with an alternative reactive agility training regime (e.g., change-of-direction training) to draw clear conclusions about the efficacy of the agility training program used in the present study on improving cognitive performance. Nevertheless, because car racing drivers continuously need to respond to several external stimuli during a race (including light displays on the steering wheel), we hypothesize that using flashing light devices that produce external stimuli to which the participants need to react may improve the reactive ability, and also the cognitive and physiological performance of car racing drivers in a higher extent as compared to other agility training regimes. Future studies should address this hypothesis via a more rigorous double-blinded randomized controlled trial also controlling for training effects. Another limitation of this study is that we determined driving performance neither in a racing environment simulator nor in real life on a racetrack. Therefore, future studies should determine changes in real driving performance in response to an intervention preferably while acquiring biosignals. Another limitation of the present study is that we did not control the nutritional intake of our participants throughout the 6-weeks reactive agility training program; therefore, it is not clear whether changes in body-fat-related anthropometric measures are the consequences of the training or an inconsistent calorie intake. Finally, although both testing and training sessions were controlled for Hawthorne effects, such placebo effects on performance gains cannot be ruled out completely.

## Conclusions

In conclusion, the reactive agility training program improved cognitive performance on some sub-tasks of the VTS when performed at rest or during moderate intensity exercise. What is more, car racing drivers performed the STROOP word-reading condition more accurately when the task was performed during the exercise vs. rest. In addition, the training intervention induced beneficial changes in some physiological measures so that peak heart rate, the heart rate at gas exchange threshold, ventilation, and relative maximal oxygen consumption increased during the maximal incremental cardio-respiratory test. Finally, car racing drivers performed the reactive agility task with improved performance and decreased reaction time throughout the 6-week training program. Overall, in line with our hypothesis, our results indicate that such a short training program that is easy to administer has the potential to contribute to induce beneficial effects on some physiological and cognitive performance measures. Therefore, it may have the potential to contribute to car racing drivers’ physical and mental preparation. Future studies should gather further scientific data on this relatively understudied sport to develop strategies that may improve motorsport athletes’ physical and cognitive abilities.

## Supplementary Information

**Additional file 1**. CONSORT Checklist.

**Additional file 2**. Placement of the 8 LED lamps during the reactive agility training program.

**Additional file 3**. Supplementary data for the results of cognitive and physiological measures in response to the reactive agility training program.

**Additional file 4**. Supplementary data for changes in performance and physiological measures throughout the training program.

## Data Availability

The datasets used and/or analyzed during the current study are presented within the manuscript and/or additional supporting files, and also available from the corresponding author on reasonable request.

## Abbreviations

ANOVA: Analysis of variance
BF: Breathing frequency
BH: Body height
BM: Body mass
CON: Control group
DT: Determination test
EXP: Experimental group
F%: Estimated fat mass
GETHR: Heart rate at gas exchange threshold
HR: Heart rate
HRR: Heart rate recovery
LVT: Visual pursuit test
M%: Estimated muscle mass
rmANOVA: Repeated measures analysis of variance
rVO_2_ max: Relative maximal oxygen consumption (adjusted to body mass)
RT: Reaction time
STROOP: Stroop test
VE: Ventilation
VISGED: Visual memory test
VCO_2_: Carbon dioxide production
VO_2_: Oxygen consumption
VTS: Vienna test system
ZBA: Time/movement anticipation test
