# Supplementary material for: Application of a Reactive Agility Training Program Using Light-Based Stimuli to Enhance the Physical and Cognitive Performance of Car Racing Drivers: A Randomized Controlled Trial
Source: Sports Med Open. 2022 Sep 5;8:113. doi: 10.1186/s40798-022-00509-9 (PMC9445110; doi:10.1186/s40798-022-00509-9)

*Supplementary material*

**Title:** Application of a reactive agility training program using light-based stimuli to enhance the physical and cognitive performance of car racing drivers

**Journal name:** Sports Medicine - Open

**Authors:** Dávid Horváth^1, 2, *^, János Négyesi^3^, Tamás Győri^2, 4^, Botond Szűcs^2, 5^, Péter János Tóth^2, 6^, Zsolt Matics^2^, Csaba Ökrös^6^, Sándor Sáfár^7^, Nikolett Szabó^8^, Beáta Takács^2^, Róbert Kathy^2^, Klára Tóth^2^, David P. Ferguson^9^, Ryoichi Nagatomi^3, 10^, Levente Rácz^1^

**Affiliations:** ^1^Department of Kinesiology, University of Physical Education, Budapest, Hungary; ^2^Fit4Race Kft., Budapest, Hungary; ^3^Division of Biomedical Engineering for Health and Welfare, Tohoku University, Sendai, Japan; ^4^Department of Psychology and Sport Psychology, University of Physical Education, Budapest, Hungary; ^5^PharmaFlight Research and Training Center, Debrecen, Hungary; ^6^Deparment of Sport Games, University of Physical Education, Budapest, Hungary; ^7^Training Theory and Methodology Research Center , University of Physical Education, Budapest, Hungary; ^8^National Academy of Handball, Balatonboglár, Hungary; ^9^Department of Kinesiology, Michigan State University, East Lansing, MI; ^10^Department of Medicine and Science in Sports and Exercise, Tohoku University Graduate School of Medicine, Sendai, Japan

**Corresponding author:**

Dávid Horváth

e-mail: david.horvath@fit4race.com

**Supplementary Figure 1:** The position of the LED lamps for the Witty SEM light stimulus.


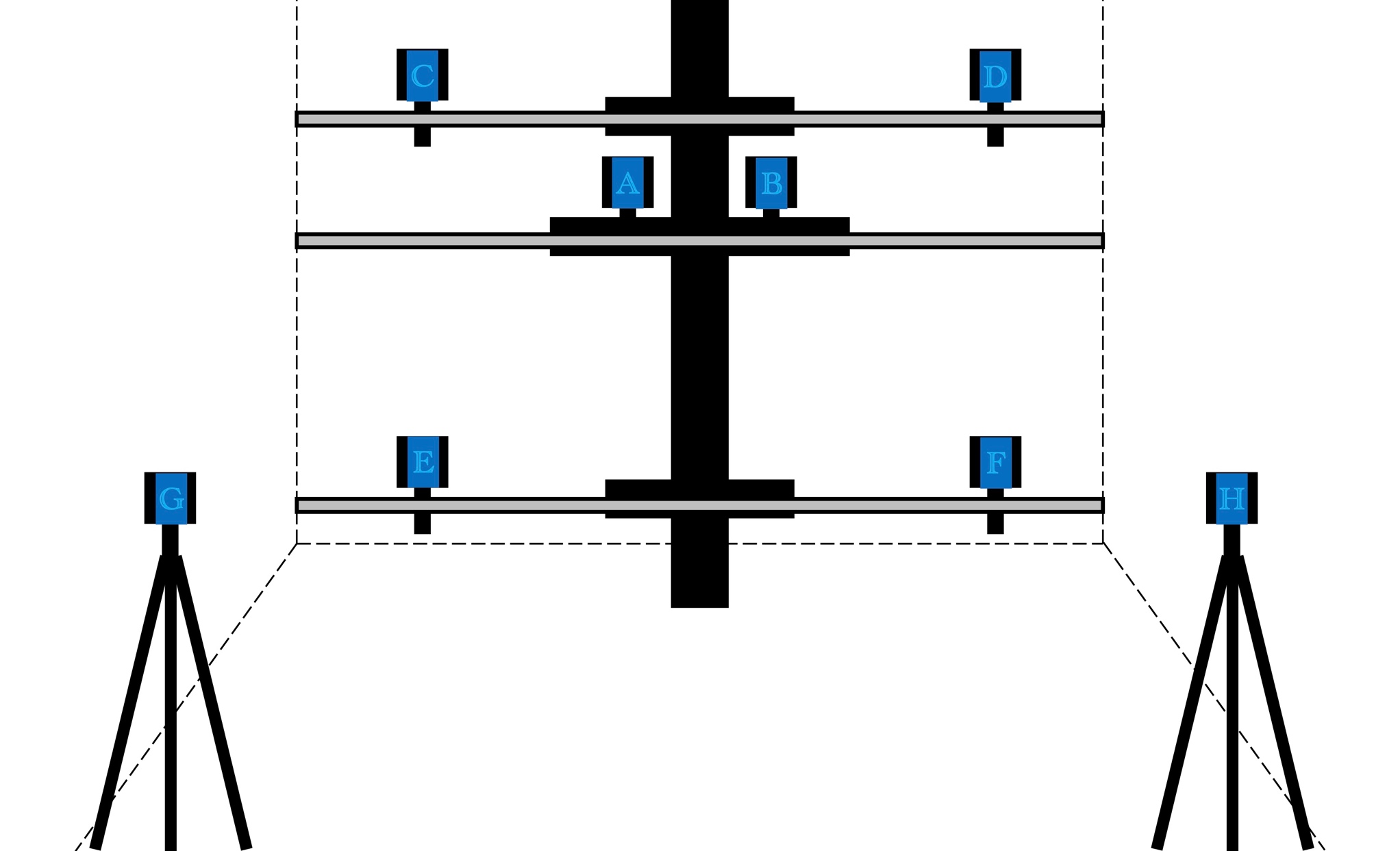

Supplement: Supplementary file 2 — Additional file 2. Placement of the 8 LED lamps during the reactive agility training program. [file 40798_2022_509_MOESM2_ESM.docx]
